# Implications of war on the food, beverage, and tobacco industry in South Korea

**DOI:** 10.1057/s41599-023-01659-1

**Published:** 2023-05-12

**Authors:** Madhusmita Bhadra, M. Junaid Gul, Gyu Sang Choi

**Affiliations:** Department of Information and Communication Engineering, Yeungnam University, Gyeongsan, Republic of Korea

**Keywords:** Economics, Economics, Business and management

## Abstract

The Food, Beverage & Tobacco (F&B) industry is an essential sector in the competitive economy. Procurement of production factors mainly depends on sales forecasting and the supply chain of raw materials. However, the conflict between Russia and Ukraine has jeopardized the global supply chain. As the conflict worsened, the world faced a food crisis, which was already a significant challenge due to the Covid-19 pandemic. Understanding how conflict-related disruptions in global food markets might affect the stock return of the F&B industry of South Korea, this study forecasts the stock returns on the KOSDAQ F&B sector. This paper highlights that the conflict resulted in immediate and far-reaching consequences on the global food supply chain and future crop harvesting in South Korea. As numerous algorithms have been widely used in predicting stock market returns, we use Autoregressive Integrated Moving Average (ARIMA) model for the prediction. Using daily returns from the KOSDAQ F&B industry from January 1999 to October 2022, the study proposes an ARIMA (2,2,3) model to forecast future movements of the stock returns. With an RMSE of 0.012, the prediction performance holds good using the ARIMA model. The results show a negative trend observed in the F&B sector returns for a few months, implying that sector stock returns decline as the conflict between Russia and Ukraine becomes more pronounced. This study also suggests that South Korea has massive scope to stabilize the demand for healthy, safe food, give more attention to domestic agribusiness, and make itself a self-sufficient agri-economy.

## Introduction

The Russia–Ukraine war, which started on February 24, 2022, put at risk the interdependence of countries worldwide (Firouzjaee and Khaliliyan, 2022). Russia has experienced severe economic distress by stringent sanctions imposed by the US, the EU, and its allies, which has caused fluctuation in the ruble’s value, a stock market crash, and a massive hike in interest rates in Russia’s domestic market (Code of Federal Regulations, 2022). These will make it more challenging for Russia to trade with the rest of the world (Orhan, 2022). Widespread fear of indiscriminate sanctions would lead Russia to defensive behavior, ultimately affecting the global supply chain (Rajan, 2022). Russia and Ukraine play a critical role in the international food market (Redeker, 2022). The Russian–Ukraine conflict comes in a challenging context, where the world is still struggling to place its economy on the betterment path after Covid-19. Amid global inflationary pressures, the Russian–Ukraine war had a negative effect on equity markets while leading to a surge in agricultural food commodities (Behnassi and El Haiba, 2022). Several commodities will see cost-push inflation (Jamil, 2022).

Russia has become a significant wheat exporter globally, ranking either first or second in wheat exports by volume from 2014 to 2020 (Wegren and Nilssen, 2022). In January 2022, South Korea imported $2.01 billion from Russia. Between January 2021 and January 2022, the imports of South Korea increased by $1.01 billion (102%) from $994 Million to $2.01 Billion (OEC—The Observatory of Economic Complexity, 2022). However, the disruption of Russia and Ukraine’s supply capacity and transportation routes throughout the conflict significantly affected the surge in prices of essential commodities for consumers in South Korea as the import was disrupted (Canuto, 2022). This paper focuses on the war’s economic implications on the South Korean food, beverage, and tobacco (F&B) sector stock market. If this war is prolonged, it might lead to galloping inflation that will impact several other sectors. There could be a spillover impact in the form of high volatility in the South Korean stock market.

This paper aims to empirically study the consequence of the Russia–Ukraine war on the performance of firms in the F&B sector. Most of the literature focused on the different factors related to a country’s economic growth (Zhou et al., 2023; Kumari et al., 2023; Izzeldin et al., 2023; Saini et al., 2023; Al-kasasbeh, 2022; Ndem et al., 2022; Aidonojie et al. 2022; Alkasasbeh and Al-kasasbeh, 2022; Saka and Abere, 2021; David, 2022; Alawi and Naho, 2022). To the best of our knowledge, there are still no empirical studies exploring the impact of the Russia–Ukraine war on the South Korean F&B sector. We focus on Korean Securities Dealers Automated Quotations (KOSDAQ) F&B sector index for our analysis. This paper, therefore, aims to fill this research gap. We use daily stock market returns from January 1999 to October 2022. Our results show a significant effect on South Korea’s F&B sectoral stock market indices over time. The results of our analysis are essentially twofold. First, this study let us understand the consequences of the ongoing conflict so that investors can manage their portfolios accordingly and policymakers can design effective financial strategies to protect themselves better. Second, our results extend earlier studies by providing new evidence by taking the case of the war in Ukraine (Hudson and Urquhart, 2022). In this paper, we focus on the F&B industry. With a motive to real-time supply chain problems, our study drives to forecast the F&B sector stock index for the investors and policy perspectives. To achieve the above-said issues, we use the Autoregressive Integrated Moving Average (ARIMA) algorithm, the best model for forecasting and modeling stock prices (Biswas, 2022; Gul et al., 2021).

The remainder of the paper is organized as follows. Section “Impact of Russia–Ukraine war on F&B industry in South Korea” discusses the impact of the Russia–Ukraine war on the F&B sector in South Korea. Section “Methodology” provides information about the dataset and the ARIMA model. Section “Forecasting” presents the forecasting of F&B stocks. Section “South Korean F&B industry in future” motivates the South Korean F&B industry in the future. Section “Conclusion” concludes the study.

## Impact of Russia–Ukraine war on F&B industry in South Korea

According to a survey of F&B manufacturers conducted in April 2020 in the United States, around 85% stated their organizations to be categorized as “essential business” following the coronavirus pandemic (Statista, 2020). This shows the significance of the F&B sector to the economy. Although various sectors are affected by the ongoing Russia–Ukraine war, we will focus on the F&B sector in this paper. The global economic situation is alarming at the outset of the conflict. The present conjuncture of restricting Russia’s imports and exports is incredibly arduous. The global economy is still exposed to the Covid-19 pandemic and has not recovered from economic instability. The Covid-19 pandemic broke supply chains and highlighted the need for a more resilient, flexible industrial model. However, disease and pandemics are not the only stressors for the global food market this time. Global climate change and drought tend to be the reason for crop failure in some places. Wars and transportation crises are factors in rising global food insecurity, requiring greater attention to transporting food from surplus points to famine places.

According to Korea Customs Service, the price of grain imports per ton in February was $386, up 26% from the same period in 2021. Compared to February 2020, just before the Covid-19 crisis began, 2022’s figure is 47.4% higher. When the global agriculture market saw steep price rises between 2007 and 2008 and 2010 and 2011, the price hikes were caused by the increased demand for bioenergy. Given that soybean and corn are primary bioenergy resources, grain prices will keep rising. Agriculture contributes about 1.8% of the gross domestic product of South Korea (Odey et al., 2022). Agri-food productivity in South Korea is confined; hence, the food supply relies on imports to a high extent (Ministry of Food and Drug Safety—Republic of Korea, 2023). With inhabitants of 51 million, South Korea is very dependent on imports for its food supply, as 60–70% is imported (Korea Economic Snapshot—OECD, 2023). The import has been increasing yearly as local agriculture and food production is insufficient to keep up with the increasing demand of its populace. According to data from the Korea Rural Economic Institute, the country’s food self-sufficiency rate has declined from 50.2% in 2015 to 48.7% in 2017 and 45.8% in 2019. Comparatively, Korea’s grain self-sufficiency rate declined to 21% in 2019 from 23.9% in 2015. Between 2016 and 2020, Korea was included in the top eight importers of Ukrainian wheat and the top three importers of Russian corn. While post-pandemic global demand, extreme weather, tightening food stocks, high energy prices, supply chain bottlenecks, and export restrictions and taxes have been straining the food market for the last two years, the recent convergence of all these factors following the Russia–Ukraine war is unprecedented and posing a threat to Korea’s food security given that South Korea accounts for around 30% of its traded wheat. South Korea, which has a low food self-sufficiency rate, could face a food crisis in the worst case due to rising overall grain prices due to the conflict and abnormal climate conditions caused by environmental change and the prolonged Covid-19 pandemic. Wheat prices will naturally rise if Ukraine and Russia experience a setback in wheat exports. In addition, corn and soybean prices, which are highly synchronized with wheat prices, will also rise, adversely affecting Korea.

**Fig. 1:**
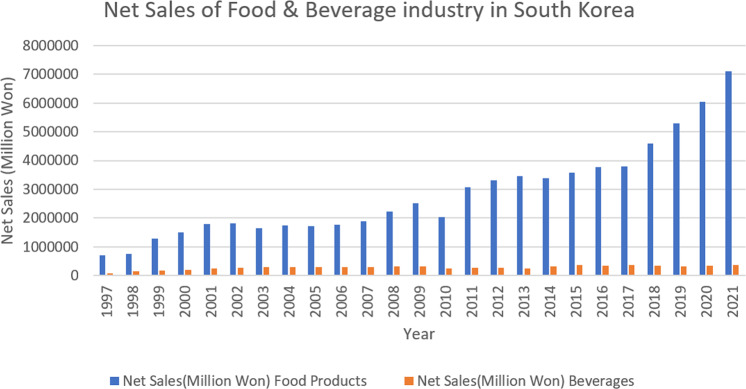
Net sales of the F&B industry in South Korea.

**Fig. 2:**
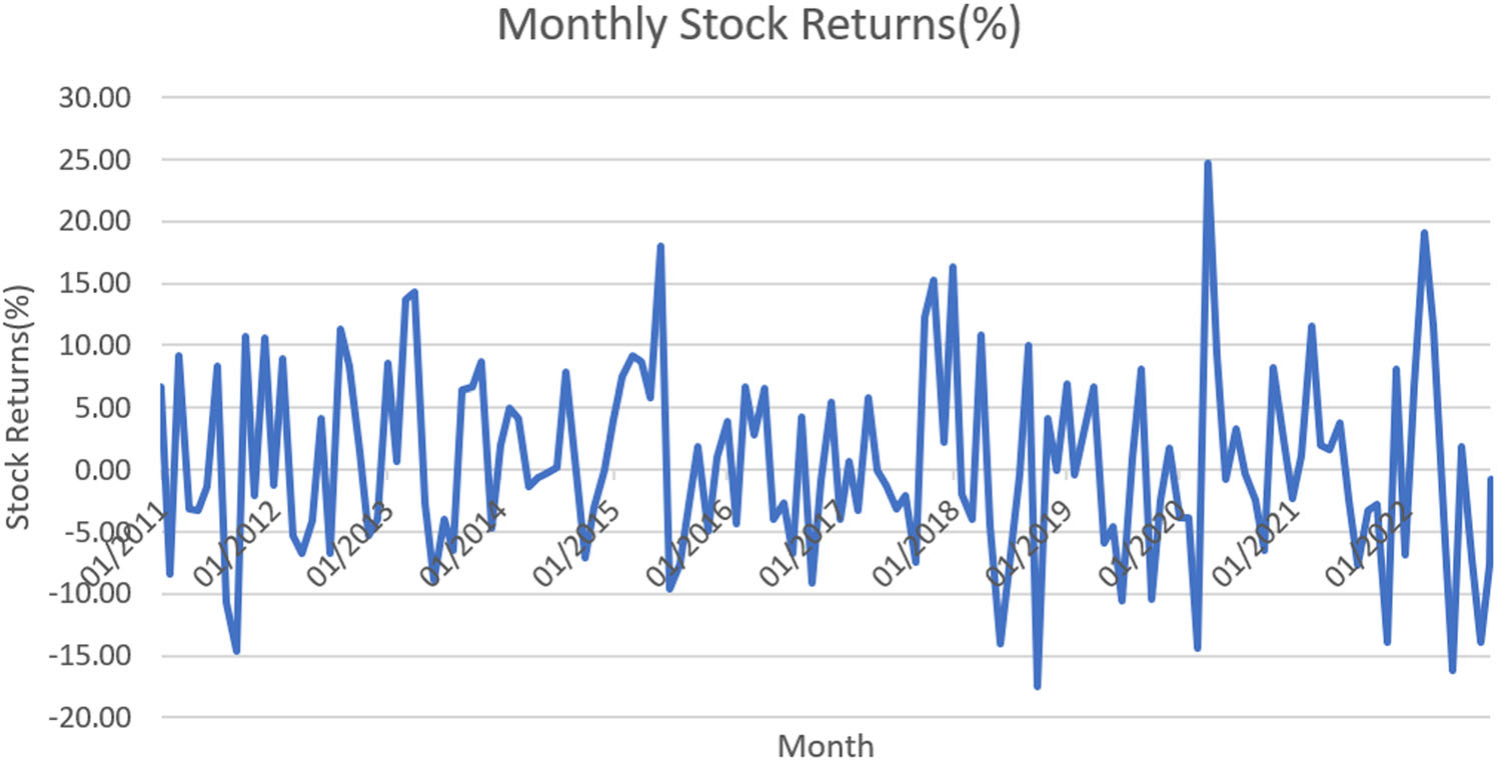
Monthly stock returns of F&B sector (%).

The Russian–Ukraine war will hit five essential commodities: energy, food, transport, metals, and microchips. Energy and commodity prices, including wheat and other grains, have increased drastically, intensifying inflationary pressures from supply chain disruptions (Canuto, 2022). South Korea’s Consumer Price Index for food has risen by more than five percent since April 2022 (Bank of Korea, 2023). Global food prices are already surging because of the increased food demand, Covid-19-related port disruptions, and massively increased shipping costs around the globe. With higher shipping costs and rerouting efforts in logistics, the impact of the Russia–Ukraine war can be expected to lead to even higher freight rates. Freight rates are directly exposed to supply-side disruptions and slowing domestic consumption or demand. Although there was an opportunity for increased demand in the F&B sector in post-covid times, because of war, F&B industries may be unable to fulfill the demand due to supply-side disruptions in sourcing raw materials and logistics challenges. The recovery scenarios are difficult to predict; however, stabilizing the domestic supply chain by improving the domestic F&B sector is possible. As per Fig. 1, we observed an increasing trend in the net sales of the F&B industry, which opened the scope for the F&B industry within South Korea. However, as per Fig. 2, a downward trend was observed in February for F&B stocks due to the Russia–Ukraine war. Our study explores the F&B stock market returns, considering the recent Ukraine crisis. While Covid-19 is the new normal, the supply chain constraints to meet the demand are the front-end challenges for the F&B industry. For the data on net sales of food products and beverages, we collected data from the Total Solutions 2000 database. For data on monthly stock returns in the F&B sector, we collected data from the DataGuide database.

## Methodology

**Fig. 3:**
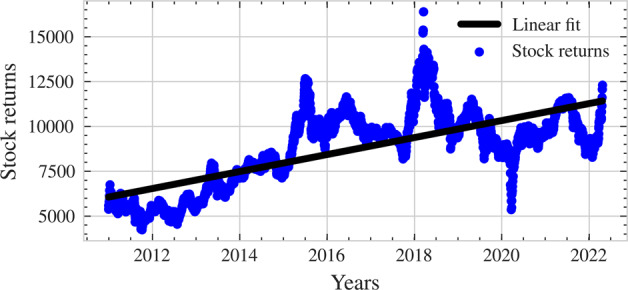
Linear fit line.

**Fig. 4:**
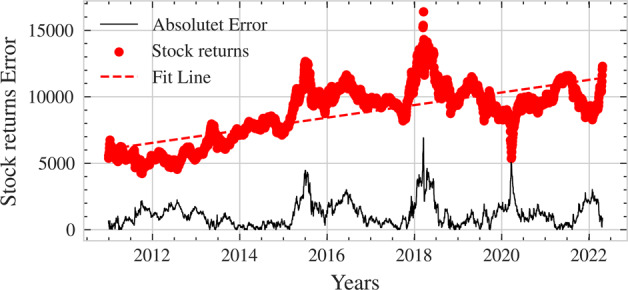
Error metric graph.

In our study, firstly, we analyzed our dataset with regression to create a base model for comparative analysis. Figure 3 shows the linear fit for the F&B stock returns. Likewise, Fig. 4 provides information about absolute error for the regression base model.

Table 1 provides the respective error metrics required to compare ARIMA model results.

**Table 1:** Baseline regression model error metric.

Mean absolute error (MAE)	0.13
Mean absolute percentage error (MAPE)	13.44%
Median absolute percentage error (MDAPE)	11.13%

Our empirical analysis used the daily stock returns (%) of the KOSDAQ F&B industry from January 1999 to October 2022. We used univariate time series forecasting. We collected the stock returns data from the DataGuide database. As per earlier works of literature, there are many determinants for stock market prediction like company characteristics, macroeconomic variables, government policies, etc. (Bhadra and Kim, 2021; He et al., 2022; Khoa and Huynh, 2022; Michael et al., 2022). However, to understand the stock market movements, most investors prefer to check the past pricing history and use it to influence their future investment decisions. There are many statistical models available that we can use to predict the stock price movement using historical stock prices. However, with the development of machine learning algorithms, prediction models gradually shifted from traditional statistical models to deep learning models (Benvenuto et al., 2020; Michalková and Pobočíková, 2023; Nand et al., 2023). The purpose of using the ARIMA model for forecasting future returns is that the historical values of the stock returns can predict future stock returns. ARIMA is a combination of autoregressive (AR) and moving average (MA) models. The mathematical formula of the model is Eq. (1)

1 $$\left( {1 - \mathop {\sum}\nolimits_{k = 1}^p {\alpha _kL^k} } \right)\left( {1 - L} \right)^dX_t = \left( {1 + \mathop {\sum}\nolimits_{k = 1}^q {\beta _kL^k} } \right)\varepsilon _t$$

where *L* is the lag operator, the *α*_*k*_ are the parameters of the autoregressive part of the model, the *β*_*k*_ are the parameters of the moving average part and *ϵ*_*t*_ are the error terms. We used Box–Jenkins ARIMA, which is known as the ARIMA (*p*, *d*, *q*) model, where *p* is the number of autoregressive (AR) terms, *d* is the number of differences taken, and *q* is the number of moving average (MA) terms.

**Fig. 5:**
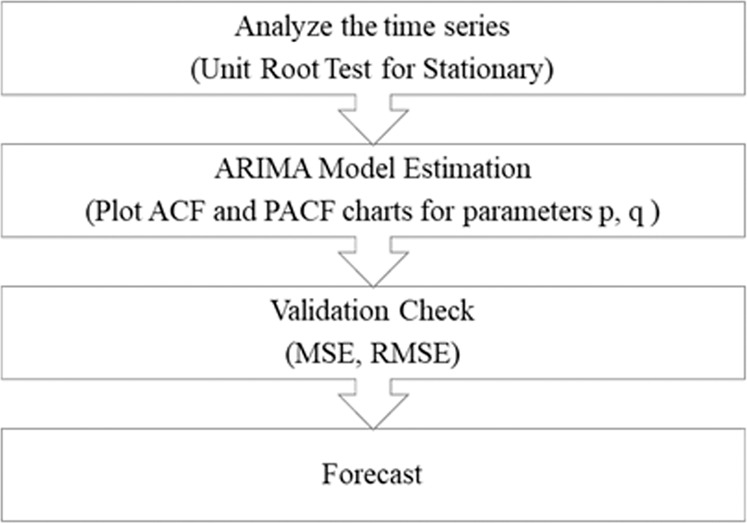
Framework for ARIMA model.

**Fig. 6:**
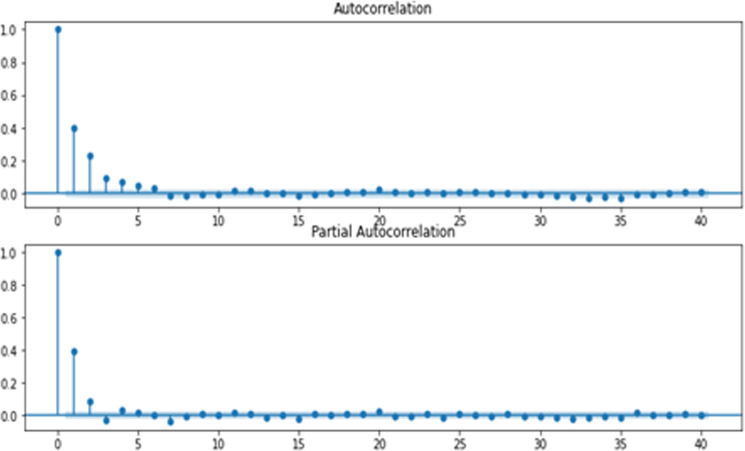
ACF and PACF.

Before applying ARIMA, we checked the stationarity of the time series. To identify the input parameters *p* and *q*, we observed the partial autocorrelation function (PACF) and autocorrelation function (ACF) charts. To determine the order of an ARIMA model, we used the Akaike information criterion (AIC) and Schwarz information criterion (SIC). The framework for ARIMA modeling is demonstrated in Fig. 5. The KOSDAQ F&B index of 8491 observations was divided into training and testing data with a ratio of 70:30, respectively. As a result, 5943 was test data, and the rest of 2548 was considered training data. The autocorrelation plot for FMCG returns is shown in Fig. 6.

Descriptive statistics help visualize the statistical features of the data before analysis. Table 2 shows the descriptive statistics of the dataset. The mean return is positive. The standard deviation of 1.8863 shows the high volatility in the F&B stock index during the sample period. Negative Skewness of −0.3847 represents an asymmetric tail which indicates a high probability of earnings from returns with high risk. The kurtosis value of the F&B stock index is 4.9737, greater than standard normal distributions, which explains the sharp peak and fat tail distribution of F&B.

**Table 2:** Descriptive statistics for KOSDAQ F&B sector.

Mean	0.073118596
Standard error	0.020471473
Median	0.11
Mode	0.05
Standard deviation	1.886377076
Sample variance	3.558418471
Kurtosis	4.973783793
Skewness	−0.384773781
Range	24.12
Minimum	−13.67
Maximum	10.45
Sum	620.85
Count	8491

The first stage of ARIMA model building is to identify the stationarity of the time series. We used Kwiatkowski–Phillips–Schmidt–Shin (KPSS) for the unit root test. The null hypothesis of the KPSS test is that the time series is non-stationary. Table 3 shows the unit root test results.

**Table 3:** Unit-Root test results for F&B Stock index returns.

KPSS statistic	0.005595947618036673
*p*-value	0.1
Num lags	31
*Critical values*	
10%	0.347
5%	0.463
2.5%	0.574
1%	0.739

The second stage of ARIMA includes the estimation of parameters *p* and *q*. Figure 6 shows the ACF and PACF plots to find the respective MA terms (*q*) and AR terms (*p*). We observed that the PACF lag 3 is significant since it is well above the significant line.

**Fig. 7:**
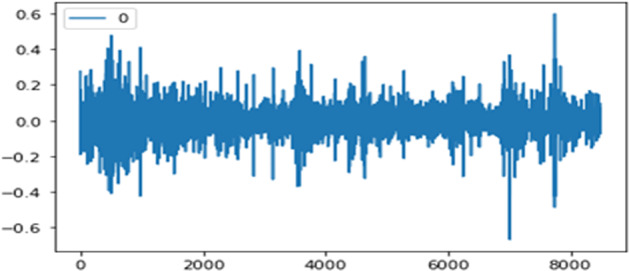
Residual errors.

**Fig. 8:**
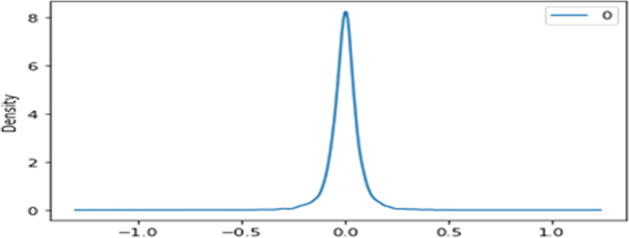
Residual errors density.

In step 3, we do a diagnostic check to validate the accuracy of the predicted values. We check if the hypotheses made on the residuals were true. We plot residuals to ensure that there are no patterns. Figure 7 shows residual errors and Fig. 8 shows the density that appeared to be fine with near-zero mean and uniform variance.

For the practical implication of a model, it is essential to check the performance of the model. For which we used different measuring forecast accuracy methods as in Table 4. RMSE of 0.012 shows the acceptance of the ARIMA model to predict stock market movements.

**Table 4:** Evaluation of forecast accuracy.

Measuring forecast accuracy methods	
RMSE	0.012
MAE	0.007
MAPE	0.015

## Forecasting

After completing the estimation phase, we attempted to forecast future stock returns by comparing them with the actual stock returns.

**Fig. 9:**
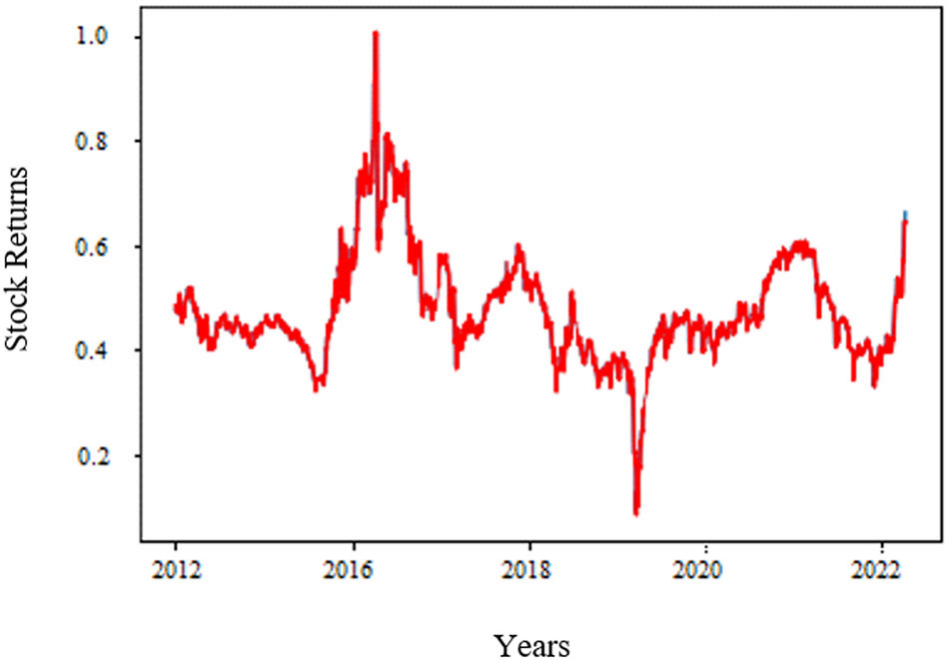
Stock forecast.

**Fig. 10:**
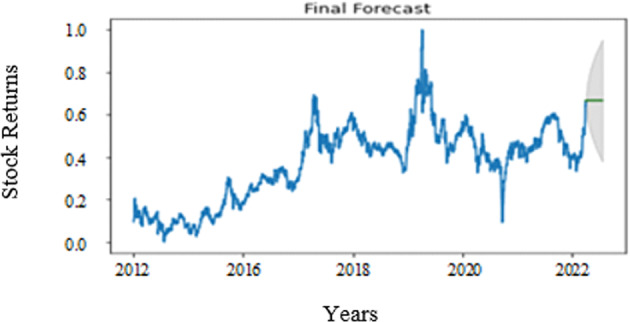
Prediction with ARIMA(2,2,3).

We used the period from January 1999 to December 2021 for training. The actual values are shown in Fig. 9 and forecast values are depicted in Fig. 10. After defining the most appropriate model, we make the forecasting of KOSDAQ F&B sector returns.

## South Korean F&B industry in future

The South Korean F&B market was valued at USD 81,638.80 million in 2021 and is projected to witness a compound annual growth rate (CAGR) of 4.63% during the forecast period, 2022–2027 (Mordor Intelligence, 2022).

South Korea mainly relies on imports for some categories of agri-food products (The Food and Beverage Market Entry Handbook—Publications Office of the EU, 2019). Dependence on imported food supplies raises the question of how South Korea’s food industry is evolving times and whether its current government policies and institutions are appropriate to achieve food security in the coming days. South Korea has a structural food deficit, and this shortage opens a market for agri-food imports to satisfy the growing consumer needs. South Korea imports more than 54.2% of its food needs which will continue to grow in the coming years. In 2020, South Korea’s agri-food and seafood trade deficit was $24.4 billion. South Korea’s agri-food and seafood imports increased by 1.0% from 2019 and a CAGR of 4.4% between 2016 and 2020 (Market Overview—South Korea—Government of Canada, 2022). Domestic agribusiness needs to be expanded to minimize the import with the increased food demand.

Traditionally, South Korean meals have centered around rice accompanied by side dishes like fish, vegetables, meat, etc. While domestic rice production is generally sufficient to meet consumer demand, there is a deficit of other ingredients. Furthermore, there is an increasing demand for unconventional imported agri-food products observed in South Korea. Data from 2010 to 2014 from the Korean Food and Drug Administration showed a substantial increase in imported processed food items. More recently, consumers have been intensely interested in imported beers. In addition, imported foods are in demand both as ingredients for traditional cuisine; and as distinct, nontraditional final products (The Food and Beverage Market Entry Handbook—Publications Office of the EU, 2019). With globalization and ease of travel and study in different parts of the globe, young consumers are increasingly exposed to foreign cultures. Hence, there is a demand for imported agri-food products to meet the demands of the diet. Single-person households in South Korea now account for a quarter of all South Korean households (OECD—Rejuvenating Korea, 2019). With the increase in single-person households and an aging population, there is an increased interest in ready-to-use food options. Furthermore, there is a culture of working long hours in South Korea. These practices of long working hours impact food and drink consumption methods. So, it is very much essential for South Korea to improve the domestic F&B industry.

**Fig. 11:**
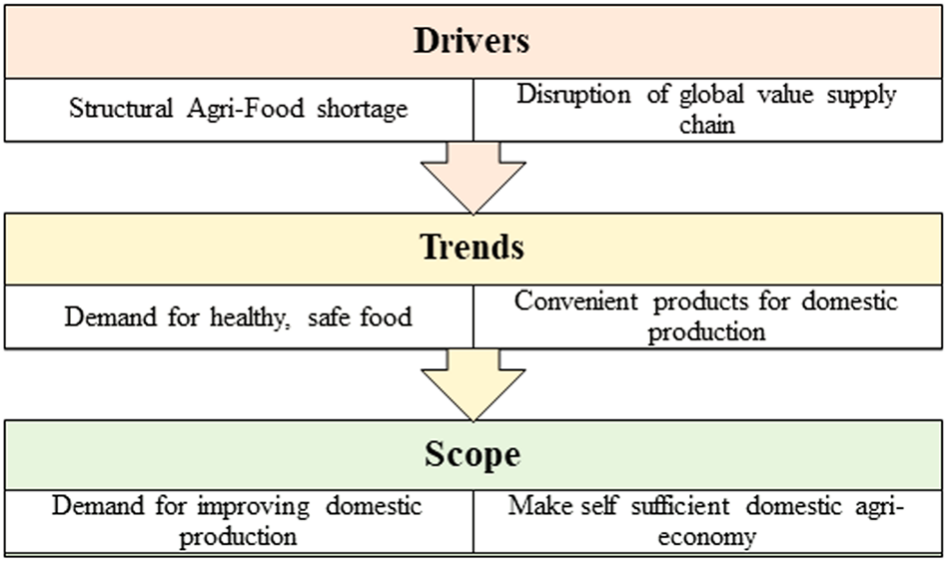
Growth framework for South Korean F&B market.

In Fig. 11, we suggested a growth framework for the South Korean F&B market where we emphasized three pillars. South Korea should implement decisive policies for the Korean agri-food industry to tackle the current economic shrink and maintain the competitiveness of the F&B industry. Considering the spread of the Covid-19 pandemic, the new policies should be implemented swiftly and boldly. The South Korean government also has attempted to promote smart agriculture by implementing the agri-food ICT (information and communications technology) policy and increasing the R&D budget by more than double in recent years (Lee et al., 2022). The South Korean government has implemented a national policy to establish smart farming communities, a concept that addresses the entire agri-food supply chain (O’Shaughnessy et al., 2021). South Korea has a very advanced modern transportation network (National transportation networks, 2022). Logistic channels in South Korea have gone through a period of transformation. Domestic players control the retail market, sometimes forming multi-layered organizations with vast organizational capacities. Furthermore, the importance of online shopping channels has significantly increased as more South Koreans consider purchasing online. In the wake of disruption in the global supply chain, South Korea has massive scope to stabilize the demand for healthy, safe food, give more attention to domestic agribusiness, and make itself a self-sufficient Agri-economy.

## Conclusion

One of the most challenging questions in recent times is the consequences of the Russia–Ukraine war on food security, which worsen by shipping constraints, energy price vulnerabilities, and inefficiencies in food production. To be self-reliant in food security, South Korea needs a great transition to strengthen the food supply chain by adopting urgent and long-term reforms and policies ranging from agriculture and forestry to industry. Our study aims to analyze the impact of the Russian–Ukraine war on the KOSDAQ stock exchange, especially in the F&B sector, and the scope of the South Korean F&B industry in the future. The prediction results show that the market has changed significantly since the Russian–Ukraine war. As a result of the analysis, we observe that the Russian–Ukraine war adversely affected the F&B sector in South Korea. Therefore, the above results show significant changes and impacts on the F&B sector during the study period. As one of the most dynamic sectors, the F&B sector needs to deal with flexible plans to deal with sudden changes in the future. The turbulence caused by the recent Russia–Ukraine war may entail beyond security challenges and may come across severe food security crises. Energy crises, shipping constraints, and climate hazards may worsen food security. In the coming days, this situation may compromise the achievements made through the years in the field of food security to decrease global hunger. For a country like South Korea, it’s crucial to feed its population, which is more than 51 million. Local food production benefits sustainable regional development and should be considered a pillar of sustainable regional development strategies. Although there is an energy crisis, shipping constraints, and climate hazards that may worsen food security, with South Korea’s advanced technology and infrastructure, it seems not so far to bolster the economy with self-reliance on food security.

Due to data scarcity, we provide the impact of war on the food supply chain in South Korea. Future studies should include other countries to corroborate our findings in addition to considering rising energy prices to capture how the war engagement significantly impacted global food security as well as energy security and how it can be avoided. In a growing complexity and uncertainty, food and energy security play an important role in achieving United Nations Sustainable Development Goals(UN SDG). We hope the world will come forward to conceptualize a possible compromise to end this conflict. Peaceful coexistence is always better than war, no matter how engaged.

## Data Availability

Our data analysis used daily stock returns (%) of the KOSDAQ F&B industry from January 1999 to October 2022. We collected the stock returns data from the DataGuide database.
